# ﻿Two new species of *Hesperopenna* Medvedev & Dang, 1981 (Coleoptera, Chrysomelidae, Galerucinae) from Singapore

**DOI:** 10.3897/zookeys.1192.116516

**Published:** 2024-02-19

**Authors:** Jan Bezděk, David Kopr

**Affiliations:** 1 Mendel University in Brno, Department of Zoology, Fisheries, Hydrobiology and Apiculture, Zemĕdĕlská 1, 613 00 Brno, Czech Republic Mendel University in Brno Brno Czech Republic

**Keywords:** Charles Fuller Baker, Leaf beetles, Oriental Region, taxonomy

## Abstract

Two new species of *Hesperopenna* Medvedev & Dang, 1981 are described from Singapore: *H.temasek***sp. nov.** and *H.bakeri***sp. nov.** The specimens of both new species were collected by Charles Fuller Baker and found in the unidentified Galerucinae material deposited in the National Museum of Natural History, Smithsonian Institution, Washington, DC. *Hesperopennatemasek***sp. nov.** is diagnosed by the black extreme elytral suture in the basal third, antennae longer than the body, the structure of the penis, and the last abdominal ventrite with two deep U-shaped incisions in females. *Hesperopennabakeri***sp. nov.** is diagnosed by the black tibia and first two tarsomeres, and the structure of the penis.

## ﻿Introduction

The genus *Hesperopenna* Medvedev & Dang, 1981 was proposed for a single species *H.flava* Medvedev & Dang, 1981 from Vietnam (Medvedev and Dang 1981). The taxonomic history of *Hesperopenna* is complicated, as the species were placed in several genera. Bezděk (2013) redefined the genus, synonymised the genera *Liroetiella* Kimoto, 1989, *Martinella* Medvedev, 2000 and *Levnma* Özdikmen, 2008 with *Hesperopenna*, and transferred species dispersed in *Calomicrus* Dillwyn, 1829, *Luperus* Geoffroy, 1762 and *Microlepta* Jacoby, 1886 to *Hesperopenna*. In the same paper (Bezděk 2013) the species of *Hesperopenna* were divided into six species groups based on the structure of male genitalia and external body characters. Meantime, additional new species were described by Medvedev (2013a, b) and Medvedev and Romantsov (2013). Bezděk (2016) revised the *Hesperopennavietnamica* species group, made additional taxonomical changes in the genus, and pointed out that the classification of species groups is insufficient and re-evaluation is necessary. Recently, one additional species from Halmahera was described (Bezděk 2023).

*Hesperopenna* species are characterised by a combination of filiform antennae, the anterior margin of pronotum unbordered, the pronotum regularly convex with a shallow oblique impression behind the anterior angles, the procoxal cavities open, the apices of the meso- and metatibiae with a spine. Additionally, metatarsomere I is about as long as two following metatarsomeres combined, the claws are appendiculate, and usually, the aedeagus has a complicated structure (Bezděk 2013, 2016). The dorsum of almost all known species is completely pale; black dorsal coloration can be found only in certain species (see Bezděk 2023). The biology and immature stages of *Hesperopenna* are unknown.

In general appearance, *Hesperopenna* species may resemble some Oriental genera/species of the section Monoleptites (e.g., *Monolepta* Chevrolat, 1836, *Ochralea* Clark, 1865, *Paleosepharia* Laboissière, 1936, etc.) but the genus can be easily distinguished by the shorter metatarsomere I (typically elongated in Monoleptites). Also, some species of *Erganoides* Jacoby, 1903 are similar mainly to smaller species of *Hesperopenna*. However, the pronotum of *Erganoides* species is regularly convex, without any oblique impression behind anterior angles.

While studying undetermined Galerucinae material borrowed from the USNM, we discovered two new species of *Hesperopenna* from Singapore. The specimens were collected by Charles Fuller Baker (1872–1927), an American entomologist, botanist and agronomist. In 1912 he moved to the Philippines and was Professor and Dean of the College of Agriculture at Los Baños. During his long stay in the Philippines, he left only once and that was for a year´s leave of absence in 1917–1918 to become assistant director of the Botanic Gardens at Singapore. According to his long-standing will, the main insect collection was bequeathed to the U.S. National Museum (Essig 1927). The type series of both new species were most likely collected in the years 1917–1918 during his stay in Singapore.

## ﻿Materials and methods

All measurements were made using an ocular grid mounted on an MBS-10 stereomicroscope (at 16× magnification for the body length and 32× magnification for the remaining measurements). Photographs of specimens were taken with a Canon 800D digital camera with a Canon MP-E 65 mm objective. Images of the same objects at different focal planes were combined using Helicon Focus 8 software. The base distribution map was downloaded from https://d-maps.com/. The pictures were edited with Corel Photopaint 12.

Specimens studied herein are deposited at the following institutes and collections:

**JBCB** Jan Bezděk collection, Brno, Czech Republic;

**USNM**National Museum of Natural History, Smithsonian Institution, Washington, DC, USA (Alexander S. Konstantinov).

Exact label data are cited for all type specimens of described species; a double slash (//) divides the data on different labels and a single slash (/) divides the data in different rows.

## ﻿Taxonomy

### ﻿ Hesperopenna temasek

Taxon classificationAnimaliaColeopteraChrysomelidae

Bezděk & Kopr sp. nov.

1AB76E68-F92E-503A-8263-220F5A7E10FE

https://zoobank.org/930B1890-E499-4A45-9687-C9B1A0A66D8B

Figs 1A–D , 2

#### Type locality.

Singapore, approx. 1°17'N, 103°51'E.

#### Type material.

***Holotype***: ♂ (USNM), “Singapore / Coll. Baker [printed white label]”. ***Paratypes***: 4 ♂♂ 5 ♀♀ (USNM, 1 ♂ 1 ♀ in JBCB), same label as holotype; 1 ♂ 1 ♀ (USNM), “Singapore / Coll. Baker [printed white label] // 17369 [handwritten white label]”. The specimens are provided with additional printed red label: “HOLOTYPUS, [or PARATYPUS] / Hesperopenna / temasek sp. nov., / J. Bezděk & / D. Kopr det. 2023 [printed red label]”.

#### Description.

Body length: ♂♂: 3.6–4.3 mm (holotype 4.3 mm), ♀♀: 3.5–4.3 mm. Body elongate oval, moderately convex, and glabrous. Body brown, except dark apices and outer basal parts of mandibles, furrows around antennal socket and frontal tubercles, extreme elytral suture in basal third, outer extreme margins of epipleura in basal third, and mesepisterna. Antennomeres brown, darkened apical part of each antennomere. Legs with slightly infuscate last two tarsomeres.

**Figure 1.**
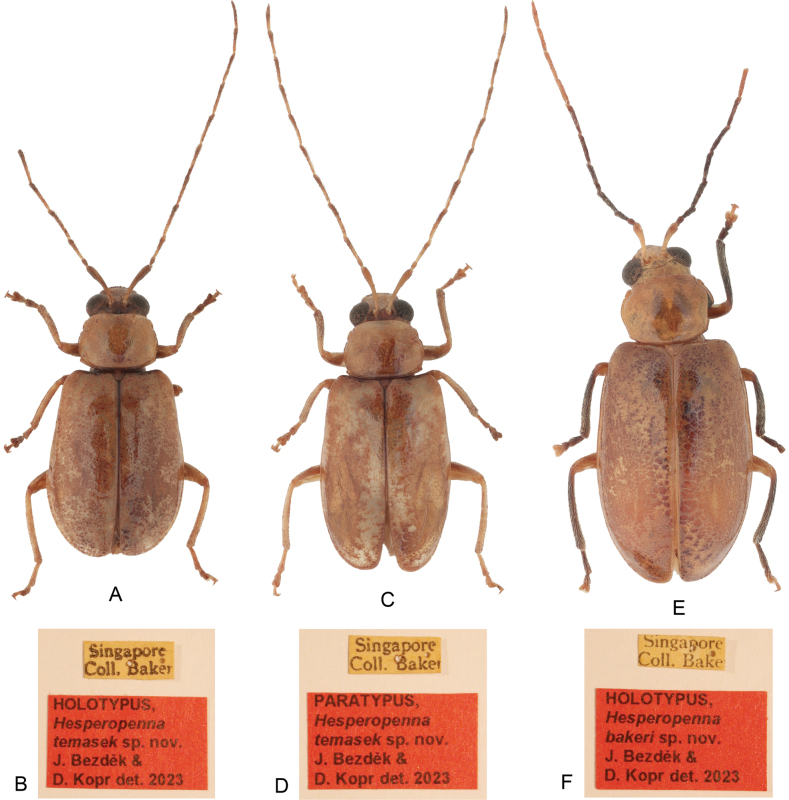
Habitus, dorsal view **A***Hesperopennatemasek* sp. nov., holotype, male **B***H.temasek* sp. nov., holotype, labels **C***H.temasek* sp. nov., paratype, female **D***H.temasek* sp. nov., paratype, labels **E***H.bakeri* sp. nov., holotype, male **F***H.bakeri* sp. nov., holotype, labels.

**Figure 2.**
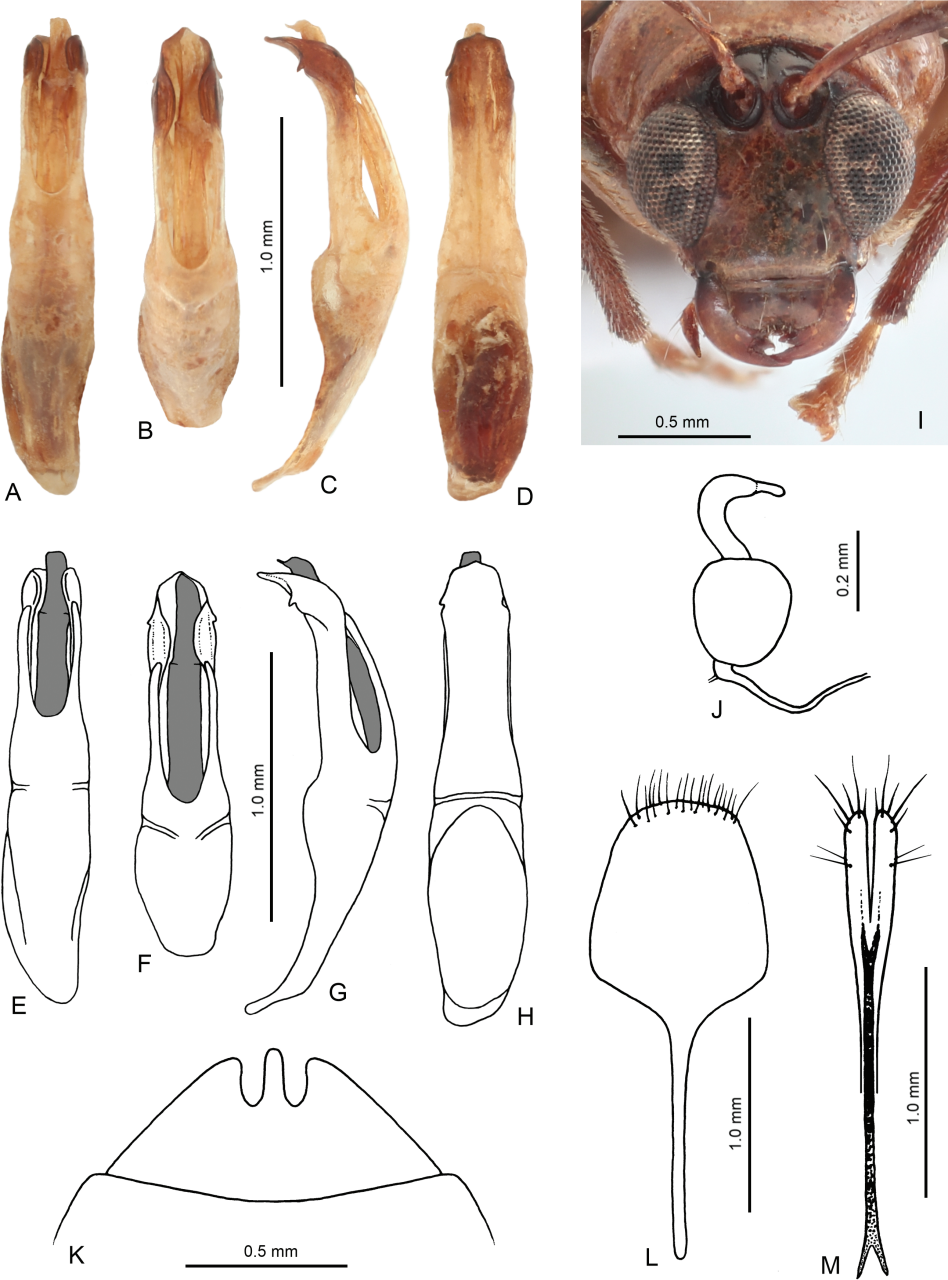
Diagnostic characters of *Hesperopennatemasek* sp. nov. **A** penis, dorsal view **B** penis, apical part **C** penis, lateral view **D** penis, ventral view **E** penis, dorsal view, drawing **F** penis, apical part, drawing **G** penis, lateral view, drawing **H** penis, ventral view, drawing **I** head **J** spermatheca **K** last visible abdominal ventrite, female **L** sternite VIII, female **M** gonocoxae.

**Male** (holotype, Fig. 1A, B). Head with transverse rectangular labrum, with rounded anterior angles, anterior margin emarginated in middle, surface with six pores in transverse row, each bearing long, pale seta. Mandibles slightly enlarged, well visible (Fig. 2I). Anterior part of head flat, lustrous and nearly glabrous, with several setae along anterior margin and close to eyes, anterior margin straight. Interantennal space extremely narrow, 0.28 times as wide as transverse diameter of antennal socket. Interocular space 1.33 times as wide as transverse diameter of eye. Frontal tubercles subtriangular, basal and oblique sides bent, moderately elevated, dull, and separated by thin, shallow groove. Vertex glabrous, lustrous, impunctate, separated from frontal tubercles by widely bent furrow. Antennae filiform, 1.21 times as long as body, length ratios of antennomeres in sequence from first equals 100-19-50-75-69-69-62-62-62-44-56 (100 = 0.8 mm). Antennomeres I–II almost glabrous, with several long setae, III–XI densely covered with short recumbent setae mixed with sparse longer setae.

Pronotum transverse, 1.47 times as wide as long, widest in middle. Surface lustrous, glabrous, densely covered with small fine punctures, moderately convex, with shallow impressions from anterior angles parallel with anterior margin. Anterior margin straight, lateral margins rounded, posterior margin widely rounded. Anterior margin unbordered, lateral and posterior margins distinctly bordered. Anterior angles swollen, posterior angles obtusangulate, pointed, each angle with setigerous pore bearing long seta. Scutellum small, triangular with rounded tip, impunctate, and glabrous.

Elytra 1.35 times as long as wide (measured at widest, in posterior third) and 0.67 times as long as body. Surface glabrous, densely covered with very fine, confused punctures. Humeral calli developed. Epipleura lustrous, glabrous, smooth, widest at anterior third, gradually narrowed towards elytral apex. Macropterous.

Procoxal cavities opened behind. Posterior margin of last abdominal ventrite widely concave, surface with distinct transverse impression along posterior margin. Abdomen covered with short sparse setae, posterior margin of last abdominal ventrite with longer setae. All legs densely covered with short recumbent setae. Apices of all tibiae with spine. Protarsomere I elongated triangular, slightly wider than small and triangular protarsomere II, length ratio of protarsomeres I–III and V equals 100-20-20-80 (100 = 0.25 mm). Mesotarsomere I elongated triangular, slightly wider than small and triangular mesotarsomere II, length ratio of mesotarsomeres I–III and V equals 100-50-75-125 (100 = 0.20 mm). Metatarsomere I long, narrow, slightly wider apically, length ratio of metatarsomeres I–III and V equals 100-29-43-57 (100 = 0.4 mm). Claws appendiculate.

Penis (Fig. 2A–H) elongate, 5.90 times as long as wide, subparallel, with slightly wider basal half. Apical half forming ventral groove-like plate and two narrow dorsolateral processes separated by large and deep U-shaped incision. In lateral view, apical part of ventral plate bent down forming curtain-like plate with very small sharp denticle. Penis with one long robust internal sclerite placed in ventral groove-like plate.

**Female** (Fig. 1C–D). Last abdominal ventrite with two deep U-shaped incisions separated by narrow subtriangular process (Fig. 2K). Apex of pygidium with small semicircular incision. Spermatheca with spherical nodulus and C-shaped cornu terminating by short narrow appendix (Fig. 2J). Sternite VIII shovel-like, with apical margin moderately rounded, with setae cumulated on and along apical margin, tignum narrow, straight, 1.13 times as long as sternite VIII (Fig. 2L). Gonocoxae long, 8.80 times as long as wide, apical third wider, subparallel, with split apex bearing several long setae, basal two third narrow, base bifurcated (Fig. 2M).

#### Differential diagnosis.

Dark color on dorsum is rare in *Hesperopenna* species and is known in a few species: head in *H.nigriceps* (Kimoto, 2004) from eastern India, head and pronotum in *H.nigricollis* (Kimoto, 1989) from Thailand, head and pronotum in some specimens of *H.bacboensis* (Medvedev, 2013) from Vietnam, head and scutellum in some specimens of *H.thailandica* (Kimoto, 1989) from Thailand, Laos and China (Yunnan), whole elytra in *H.gilolo* Bezděk, 2023 from Halmahera, and, finally, elytra with a black extreme lateral margin in basal half in *H.zofka* Bezděk, 2013 from Indonesia (Java, Bali). *Hesperopennatemasek* sp. nov. has black frontal tubercles and furrows around antennal sockets, extreme elytral suture in basal third, outer extreme margins of epipleura in basal third, and mesepisterna. The antennae are c. 1.20 times as long as body. Most of *Hesperopenna* species have antennae slightly shorter than body or at least slightly longer (c. 1.05 times as long as body). Longer antennae are known in *H.pallida* species group (sensu Bezděk 2013), however this group is awaiting revision. Nevertheless, the penis of *H.temasek* sp. nov. with two narrow dorsolateral processes separated by large and deep U-shaped incision and with very small sharp denticles on apicolateral curtain-like plates, is completely different in comparison with very simple structure of penis in *H.pallida* species group. Mandibles (Fig. 2I) are somewhat enlarged, better visible than in other *Hesperopenna* species.

The females are characterized by the shape of last abdominal ventrite with two deep U-shaped incisions separated by narrow subtriangular process (Fig. 2K). The females of the vast majority of *Hesperopenna* species have the posterior margin of the last abdominal ventrite entire or with one more or less shallow median emargination. The only species with similarly structured last abdominal ventrite in females is *H.nigricollis* (Kimoto, 1989) from Thailand.

#### Distribution.

Singapore.

#### Etymology.

Temasek is an early recorded name of a settlement on the site of modern Singapore. Noun in apposition.

### ﻿ Hesperopenna bakeri

Taxon classificationAnimaliaColeopteraChrysomelidae

Bezděk & Kopr sp. nov.

7A9F95B9-86E6-5D7D-A326-E8E55CE771C3

https://zoobank.org/12478282-4E4D-4385-B1FC-3782FCABCBBE

Figs 1E–F , 3

#### Type locality.

Singapore, approx. 1°17'N, 103°51'E.

#### Type material.

***Holotype***: ♂ (USNM), “Singapore / Coll. Baker [printed white label]”. ***Paratypes***: 2 ♀♀ (USNM), same label as holotype. The specimens are provided with additional printed red label: “HOLOTYPUS, [or PARATYPUS] / Hesperopenna / bakeri sp. nov., / J. Bezděk & / D. Kopr det. 2023 [printed red label]”.

#### Description.

Body length: ♂: 5.6 mm (holotype), ♀♀: 5.1–5.8 mm. Body elongate oval, moderately convex, and glabrous. Body orange brown, except darkened apices of mandibles. Antennomeres I–II orange, III–VI black, VII dark brown, VIII–XI brown. Legs brown with black tibia and first two tarsomeres.

**Male** (holotype, Fig. 1E–F). Head with transverse rectangular labrum, with rounded anterior angles, anterior margin straight and shallowly emarginated in middle, surface with six pores in transverse row, each bearing long, pale seta. Anterior part of head slightly convex, lustrous and nearly glabrous, with several setae along anterior margin and close to eyes, anterior margin slightly concave. Interantennal space narrow, 0.71 times as wide as transverse diameter of antennal socket. Interocular space 1.35 times as wide as transverse diameter of eye. Frontal tubercles transverse, outer parts narrow and transverse, subtriangular medially, moderately elevated, lustrous, and separated by thin, shallow groove. Vertex glabrous, lustrous, impunctate, separated from frontal tubercles by shallow bent line. Antennae filiform, 0.80 times as long as body, length ratios of antennomeres in sequence from first equals 100-26-52-87-87-87-87-83-83-69-83 (100 = 0.6 mm). Antennomeres I–II almost glabrous, with several long setae, III–XI densely covered with short recumbent setae mixed with sparse longer setae.

Pronotum transverse, 1.35 times as wide as long, widest in middle. Surface lustrous, glabrous, covered with indistinct punctures, moderately convex, with shallow impressions from anterior angles parallel with anterior margin. Anterior margin straight, lateral margins rounded, posterior margin moderately rounded. Anterior margin unbordered, lateral and posterior margins distinctly bordered. Anterior angles distinctly swollen, posterior angles obtusely angulate, each angle with setigerous pore bearing long seta. Scutellum small, triangular with rounded apex, impunctate, and glabrous.

Elytra 1.60 times as long as wide (measured at widest, in posterior third) and 0.71 times as long as body. Surface glabrous except very scarce short setae on apical slopes and on lateral and apical margins, densely covered with very small, confused punctures. Humeral calli developed. Epipleura lustrous, glabrous, smooth, widest at anterior third, gradually narrowed towards elytral apex. Macropterous.

**Figure 3.**
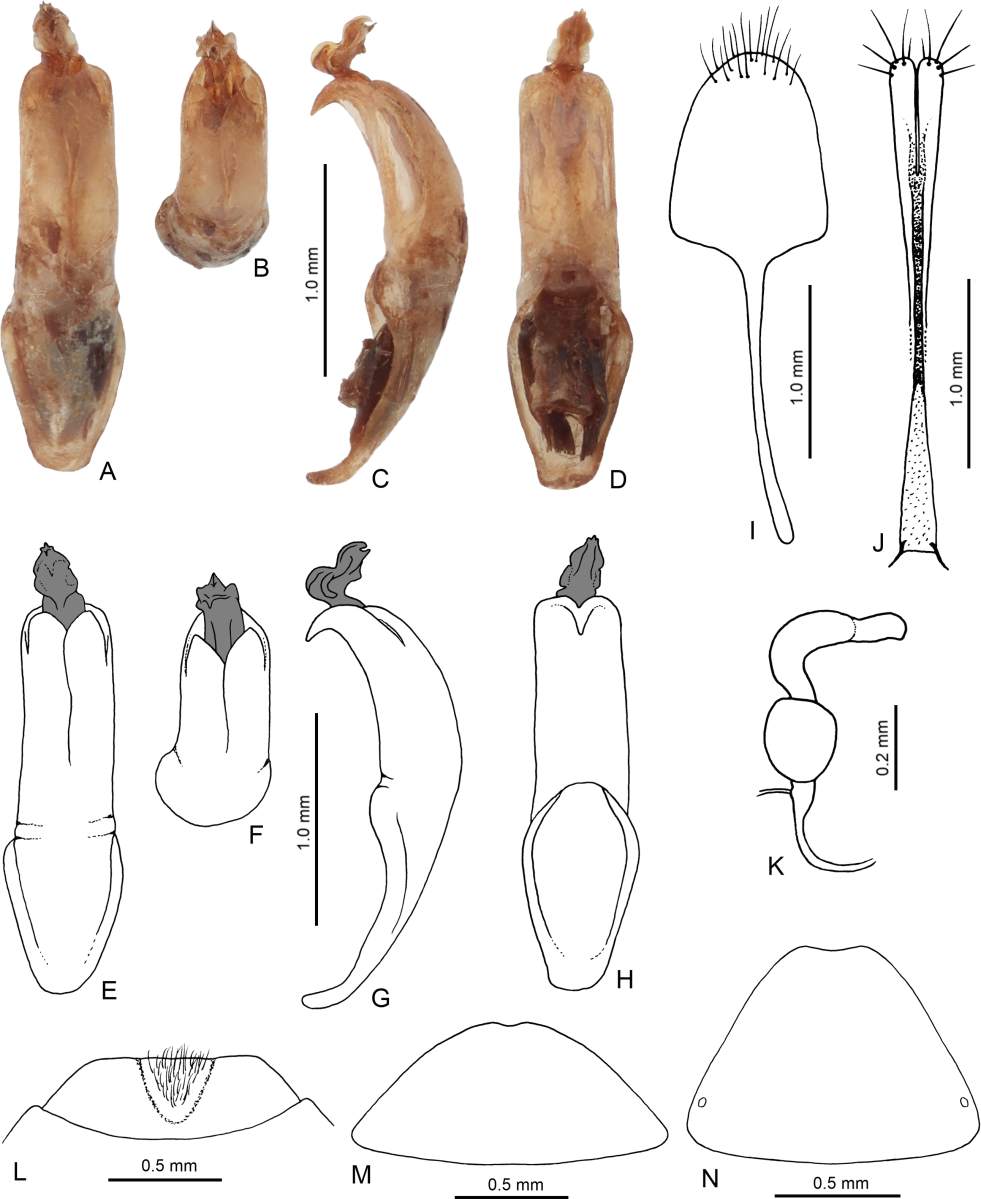
Diagnostic characters of *Hesperopennabakeri* sp. nov. **A** penis, dorsal view **B** penis, apical part **C** penis, lateral view **D** penis, ventral view **E** penis, dorsal view, drawing **F** penis, apical part, drawing **G** penis, lateral view, drawing **H** penis, ventral view, drawing **I** sternite VIII, female **J** gonocoxae **K** spermatheca **L** last visible abdominal ventrite, male **M** last visible abdominal ventrite, female **N** pygidium, female.

Procoxal cavities open behind. Last abdominal ventrite with well visible impressed furrows forming subtriangular plate, posterior margin of last abdominal ventrite nearly straight. Abdomen covered with sparse setae, plate on last abdominal ventrite with longer and denser setae (Fig. 3L). All legs densely covered with short recumbent setae. Apices of meso- and metatibiae with spine. Protarsomere I elongated subtriangular, slightly wider than small and triangular protarsomere II, length ratio of protarsomeres I–III and V equals 100-66-66-111 (100 = 0.20 mm). Mesotarsomere I elongated triangular, as wide as triangular mesotarsomere II, length ratio of mesotarsomeres I–III equals 100-50-41 (100 = 0.25 mm) (mesotarsomere V missing). Metatarsomere I long, narrow, slightly wider apically, length ratio of metatarsomeres I–III and V equals 100-41-35-65 (100 = 0.4 mm). Claws appendiculate.

Penis (Fig. 3A–H) elongate, parallel, 3.90 times as long as wide, dorsal side with two partly overlapping plates anteriorly forming two widely rounded processes. Apex triangular, strongly bent downwards. In lateral view, penis moderately bent. Penis with one long robust internal sclerite with apex bent upwards and covered with complicated structure.

**Female.** Last abdominal ventrite without impressed furrows forming subtriangular plate, posterior margin widely rounded with very small apical emargination (Fig. 3M). Apex of pygidium with wide shallow emargination (Fig. 3N). Spermatheca with spherical nodulus and C-shaped cornu, narrowed basally, terminated by wide appendix (Fig. 3K). Sternite VIII shovel-like, with widely rounded apical margin, with setae cumulated on and along apical margin, tignum narrow, slightly bent, 1.33 times as long as sternite VIII (Fig. 3I). Gonocoxae long, 9.50 times as long as wide, distinctly narrowed in middle part, with split apex, apical part with several long setae, base with two short thin processes (Fig. 3J).

#### Differential diagnosis.

Having brown legs with black tibia and first two tarsomeres *Hesperopennabakeri* sp. nov. is similar to *H.tibialis* (Kimoto, 1989) from Laos, Thailand and Peninsular Malaysia, and *H.zofka* Bezděk, 2013 from Indonesia (Java, Bali) from *Hesperopennamedvedevi* species group (see Bezděk 2013), and also to *H.vietnamica* (Medvedev, 2000), and some specimens of *H.thailandica* (Kimoto, 1989) with black tibia from *Hesperopennavietnamica* species group (see Bezděk 2016). *Hesperopennatibialis* and *H.zofka* are large species with body length more than 6.8 mm while the body length of *H.bakeri* sp. nov. is 5.6–5.8 mm. *Hesperopennabakeri* sp. nov. has less transverse pronotum, 1.35 times as wide as long, while pronota of *H.vietnamica* and *H.thailandica* are more transverse, 1.75–1.85 times as wide as long. Penis of *H.bakeri* sp. nov. (Fig. 3A–H) has dorsal side with two wide partly overlapping plates, the apex is triangular, strongly bent downwards, and endophallic sclerite is robust. Penis of *H.tibialis* and *H.zofka* is robust, with two endophallic sclerites (one very large, with spoon-like apex and distinct ridges ventrally, second thin, usually hidden inside the aedeagus – see figs in Bezděk 2013), and that of *H.vietnamica* and *H.thailandica* has two thin lateral processes with very deep incision between them, ventral side of penis apically with hook-like process, and endophallic sclerite thin (see figs in Bezděk 2016).

#### Distribution.

Singapore.

#### Etymology.

Dedicated to Charles Fuller Baker (1872–1927), an American entomologist, botanist and agronomist, who collected the type series.

## ﻿Discussion

**Figure 4.**
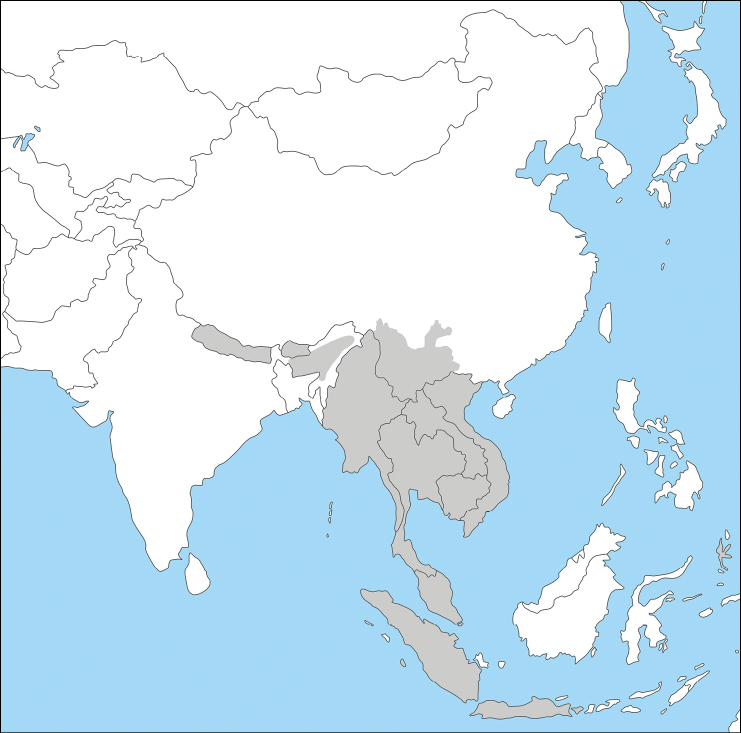
Distributional map of the genus *Hesperopenna*. The countries and regions with confirmed occurrence are colored in grey.

*Hesperopenna* currently includes 39 species (Nie et al. 2017; Bezděk 2023, present paper). Most known species occur in continental Southeast Asia (Vietnam, Laos, Thailand, Myanmar, Eastern India, and continental Malaysia). Only four species extend into the southernmost parts of the Palearctic Region (Nepal, Bhutan and Yunnan). The occurrence of *Hesperopenna* in the Sunda Islands is very little studied. Two species are known from Sumatra and one from Java and Bali, but it is assumed that there will be many more species in the Sunda Islands. *Hesperopenna* species are also confirmed in the Philippines and the island of Borneo, with several hitherto undescribed species awaiting the descriptions. The easternmost record of *Hesperopenna* is from Halmahera in the Maluku archipelago (Bezděk 2023). For a distributional map of the genus *Hesperopenna* see Fig. 4.

The depositories of large museums usually contain a large amount of unprocessed and undetermined material (Löbl 2023), often from biotopes that have completely changed their character over the years or disappeared outright. The discovery of more than a hundred-year-old specimens of two new species in museum material is not surprising. Eleven years ago, the average time span between species discovery and description was found to be 21 years (Fontaine et al. 2012), however, it is significantly longer for some insects (Löbl 2023). The existence of completely new species in the depository clearly underlines the importance of natural history museum collections for preserving evidence of global biodiversity. We can only hope that both newly described species will also be re-discovered in the wild, and that this paper does not describe species that are new to science but already extinct (compare e.g., Rossini et al. 2021).

## Supplementary Material

XML Treatment for Hesperopenna temasek

XML Treatment for Hesperopenna bakeri

## ﻿Additional information

### Author ORCIDs

Jan Bezděk https://orcid.org/0000-0003-4358-7211

David Kopr https://orcid.org/0000-0003-2466-9609

### Data availability

All of the data that support the findings of this study are available in the main text.
